# Maternal cancer and autism spectrum disorder: Analytical cross-sectional study

**DOI:** 10.1192/j.eurpsy.2021.1152

**Published:** 2021-08-13

**Authors:** A. Daldoul, R. Ben Moussa, W. Krir, S. Bouslah, H. Kefi, S. Zaied, N. Gaddour

**Affiliations:** 1 Medical Oncology, Fattouma Bourguiba University Hospital, Monastir, Tunisia; 2 Child And Adolescent Psychiatry, Fattouma Bourguiba University Hospital, Monastir, Tunisia; 3 Psychiatry, Military Hospital, Tunis, Tunisia; 4 Child And Adolescent Psychiatry Department, Fattouma Bourguiba University Hospital, Monastir, Tunisia, Monastir, Tunisia

**Keywords:** burn out, oncology, autism

## Abstract

**Introduction:**

Autism spectrum disorder (ASD) and cancer are two pathologies that appear to be different, but whose multifactorial determinism involves shared genetic and environmental factors.

**Objectives:**

This study aimed to find an association between maternal cancer and ASD in children by comparing the frequencies of ASD between children of mothers followed for cancer and children of mothers free from cancer

**Methods:**

We recruited, between March and August 2018, women divided into 2 groups: G1; women followed for cancer at the oncology consultation of Monastir and Sousse (N1 = 100) and G2; women free from cancer (N2 = 101). We screened their offspring aged 18 months to 11 years for ASD using the C-AQ and the M-CHAT. We confirmed the diagnosis of ASD using ADI-R in children who tested positive.

**Results:**

Nous avons examiné un total de 332 enfants. La fréquence des enfants atteints de TSA chez les mères G1 était de 5,4%, celle des enfants de mères G2 était de 1,2 avec une association significative entre cancer maternel et TSA (p = 0,035), qui est restée significative après analyse multivariée. (p = 0,001) D’autres facteurs que nous avons trouvés associés aux TSA dans notre population sont la présence d’une expérience stressante pendant la grossesse; exposition précoce avant l’âge de 2 ans à la télévision> 2 heures par jour; antécédents psychiatriques de la mère, du père et de la fratrie et dépression post-partum.

**Conclusions:**

Le cancer maternel et les TSA semblent partager des mécanismes étiopathogènes communs

